# ATF3 triggers M2 macrophage polarization to protect against pulp inflammation through WNT4 regulation: ATF3 triggers M2 macrophage polarization

**DOI:** 10.3724/abbs.2025005

**Published:** 2025-01-10

**Authors:** Liu Liu, Jie Wang, Jie Yu, Jing Wang, Jinhua Yu

**Affiliations:** Department of Endodontics the Affiliated Stomatological Hospital of Nanjing Medical University; Jiangsu Province Key Laboratory of Oral Diseases; Jiangsu Province Engineering Research Center of Stomatological Translational Medicine; State Key Laboratory Cultivation Base of Research Prevention and Treatment for Oral Diseases Nanjing Medical University Nanjing 210000 China

**Keywords:** ATF3, WNT4, macrophage polarization, pulpitis

## Abstract

Pulpitis is a common inflammatory oral disease that can lead to pulp necrosis. The aim of this study is to investigate the expression and regulatory mechanisms of ATF3, a potential therapeutic marker, in pulpitis. A mouse pulpitis model with different degrees of inflammation is established, and the expression of ATF3 in pulpitis is explored. The histological features of healthy pulp and pulpitis are analyzed by HE staining, and classical inflammatory factors are detected by immunohistochemistry (IHC). In an *in vitro* study, we investigate the role of ATF3 in the regulation of WNT4 transcription and explore the effects of the ATF3/WNT4 axis on the polarization of RAW264.7 macrophages, the inflammatory response and the osteogenic differentiation of human dental pulp stem/stromal cells (hDPSCs). Our results show that ATF3 is expressed at low levels in inflamed pulp tissues; overexpression of ATF3 reduces the area of pulp necrosis, decreases the level of pro-inflammatory factors, and promotes macrophage polarization toward the M2 type. Furthermore, we reveal that ATF3 binds to the WNT4 promoter region and positively regulates the expression of WNT4 and that ATF3 downregulates M1 markers and increases the expression of M2 markers by regulating WNT4 expression. In addition, ATF3 promotes the osteogenic differentiation of dental pulp stem cells. In summary, this study reveals that ATF3 promotes M2 macrophage polarization by regulating WNT4, which in turn inhibits pulpal inflammatory responses and promotes the osteogenic differentiation of dental pulp stem cells. These findings suggest that ATF3 may be a potential target for pulpitis treatment.

## Introduction

Pulpitis is an inflammatory oral disease that typically occurs in pulpal tissues and is caused by caries, trauma, and chemical irritation, with caries-borne bacterial infection being the most common etiological factor [1]. Caries is one of the most prevalent diseases worldwide, with recent reports indicating that more than 90% of adults suffer from caries [2]. Bacterial invasion of the dental pulp is a dynamic process of inflammatory injury resulting from direct bacterial damage to virulence factor damage to activate the immune response [3]. Pulpitis usually progresses from a reversible to an irreversible stage, and even in the early stages of microbial infection, bacterial components can spread through dentin tubules and cause a localized inflammatory response in the pulp [4]. If left untreated, pulpitis can progress to pulp necrosis and even severe apical periodontitis [5]. In addition, oral inflammation is a common factor that induces other systemic diseases, such as cardiovascular disease and diabetes mellitus, which seriously affect people′s quality of life and general health [6]. Therefore, the pathogenesis and treatment of endodontic inflammation has been a hot topic of research, so it is crucial to explore the molecular mechanism of endodontic inflammation development to study and find therapeutic targets.

The inflammatory response is an important defense mechanism that stimulates the repair of damage to pulpal tissues and alleviates the degree of inflammation and tissue damage [7]. Among them, the role of macrophages in pulpitis has received increasing attention. Macrophages, as remarkable plastic cells, can be transformed into different subtypes of cells, such as M1-type macrophages with pro-inflammatory effects and M2-type macrophages with anti-inflammatory effects, and these two specific phenotypes are the functional responses of macrophages to stimuli and signals released from the microenvironment in which they are located, a process referred to as macrophage polarization [ 8, 9] . M2-type macrophages play a significant role in the development and repair of endodontic tissues in the presence of Th2 lymphocytes, IL-4, IL-13, TLRs, and glucocorticoids; exhibit only weak antigen-presenting capacity; and downregulate the expression of immune responses by secreting inhibitory cytokines (IL-10, TGF-β, IL-RA, *etc*.), thereby regulating the balance of the body′s immunity [10]. In the late inflammatory response, M2-type macrophages can stimulate the proliferation and survival of major inflammatory immune cells in dental pulp tissues through immune modulation, exerting the immunomodulatory effects of phagocytosis and killing, antigen presentation, and cytokine secretion in the pulp microenvironment and promoting the repair and regeneration of local tissues in the organism [11]. Therefore, the regulation of macrophages in the dental pulp microenvironment facilitates the repair of damage to dental pulp tissues.

In the present study, we found for the first time that the activating transcription factor ATF3 was aberrantly expressed in pulpitis and that the ATF3/WNT4 axis promoted the osteogenic differentiation of pulp stem cells by modulating the polarization and inflammatory response of M2 macrophages. Our findings suggested that ATF3 is a potential target for pulpitis treatment.

## Materials and Methods

### Cell isolation and culture

Human dental pulp stem/stromal cells (hDPSCs) were isolated from normal human molar teeth as previously described. hDPSCs were incubated in Dulbecco’s modified Eagle’s medium (DMEM; #11965092; Gibco, Carlsbad, USA) supplemented with 10% fetal bovine serum (FBS; #10091148; Gibco) and 1% penicillin-streptomycin (#15140122; Gibco) in an incubator with 5% CO _2_. RAW264.7 macrophages purchased from ATCC (#TIB-71; Manassas, USA) were cultured in DMEMsupplemented with 10% FBS to provide nutrients and 1% penicillin-streptomycin solution to prevent contamination. 293T cells purchased from ATCC (#CRL-3216) were grown in DMEM supplemented with 10% FBS and 1% penicillin-streptomycin solution. The relevant information is shown in Supplementary Table S1.

### Cell treatment

The cell treatments included M1 polarization, M2 polarization and osteogenic differentiation studies of hDPSCs. RAW264.7 macrophages were treated with 100 ng/mL LPS (#L5293, purified from *Escherichia coli*; Sigma-Aldrich, St Louis, USA) for 24 h to induce M1 polarization, and 10 ng/mL IL-4 (#574302; Biolegend, San Diego, USA) was used to treat RAW264.7 macrophages for 24 h to induce M2 polarization. Osteogenic differentiation of hDPSCs was performed by transfecting RAW264.7 cells with a lentiviral vector, and the cell supernatant was collected after LPS stimulation and added to hDPSCs for culture.

### Construction of a mouse model of pulpitis

Six-week-old C57BL/6 mice (20–25 g) were purchased from the Animal Core Facility of Nanjing Medical University (Nanjing, China) and were given access to a normal diet and water for one week to prevent stress. The modelling procedure was as follows: mice were anaesthetized by intraperitoneal injection of 1% pentobarbital (#P3761; Sigma-Aldrich), placed in the supine position, and their mouths were propped open to expose the surgical area. Then, the mice were sterilized under an oral surgical microscope, and the dental tissues in the central fossa of the first molar were removed with a high-speed turbine. Upon reaching the deeper layers of the tooth, the pulp was exposed by penetrating the spinal cord with a 15# K file. The mice were divided into control groups and exposed for 12, 24 or 72 h according to the different groups ( *n *= 6). All animal experimental protocols were confirmed by the Ethics Committee of the Affiliated Stomatological Hospital of Nanjing Medical University.

### ATF3-treated model mice

The mice were divided into four groups: sham + AAV-NC, sham + AAV-ATF3, pulpitis (72 h) + AAV-NC, and pulpitis + AAV. Adeno-associated viruses (AAVs) carrying the indicated vectors were designed and synthesized by GeneScript (Nanjing, China). AAVs carrying the NC or ATF3 overexpression vector (10 μL, 2 × 10 ^9^ genomic particles in PBS) were injected intraperitoneally for 2 days prior to the operation and every day thereafter.

### Immunohistochemistry (IHC)

Pulp tissue was treated with xylene and ethanol to confer H _2_O _2_-blocking enzyme activity. The antigens were extracted with sodium citrate buffer for recovery. The samples were incubated with the antibodies shown in Supplementary Table S2. The resulting images were observed under a microscope (Olympus, Tokyo, Japan) and the stained cells and staining intensity were quantified via ImageJ software.

### Hematoxylin-Eosin (HE) staining

The pulp tissue was branched into 2-μm thick sections, baked, dewaxed in water (decarbonized in xylene and dehydrated in ethanol) and then stained with hematoxylin (#BSBA-4021; Beijing Zhong Shan-Golden Bridge Biological Technology, Beijing, China) for 10 min. Subsequently, the tissue sections were subjected to eosin staining (#318906; Sigma-Aldrich), dehydration, transparency, sealing, observation with a microscope, analysis and acquisition of the images obtained. The area of necrotic areas was determined via ImageJ software (NIH, Bethesda, USA).

### qRT-PCR analysis

Total RNA is extracted from tissues or cells using an extraction kit (#12183555; Invitrogen, Carlsbad, USA), the concentration is measured, and inputs are calculated. Reverse transcription reagents were added to immediately transcribe the cDNA, which was amplified using a StepOnePlus PCR instrument (#4376600; Applied Biosystems, Foster City, USA) and a PrimeScript RT-PCR Kit (#RR014A; Takara, Tokyo, Japan), and the quantitative results were output. Gene expression was detected via the 2 ^–ΔΔCt^ method and normalized to that of *GAPDH*. The sequences of primers are shown in Supplementary Table S3.

### Western blot analysis

Tissues or cells were lysed with RIPA lysis buffer (#89901; Thermo Fisher Scientific, Waltham, USA) to extract total protein, and the protein concentration was determined via a Pierce BCA protein assay kit (#A55864, Thermo Fisher Scientific). Denatured proteins were separated by polyacrylamide microgels. Proteins were transferred wet to a nitrocellulose membrane (Millipore, Billerica, USA) and blocked with TBST buffer containing 5% skim milk for 1 h. The membranes were incubated with specific primary antibodies at 4°C overnight and then cultured with secondary antibodies for 1 h at room temperature, followed by the addition of a color development solution (#34580; Thermo Fisher Scientific), and then an imaging system (iBright CL750, #A44116; Invitrogen) was used to display the labelling correctly. Protein quantification was conducted using ImageJ software. The information of the antibodies used is shown in Supplementary Table S2.

### Lentivirus preparation and cell transfection

The amplification primers for ATF3 and hairpin primers ( Supplementary Table S3) for WNT4 were designed by GenScript, annealed and inserted into the GV358 and pLentiGuide-Puro vectors. 293T cells were cultured to reach 70%–80% confluence. The lentiviral particles were produced by transducing the constructed vectors with the lentiviral packaging vectors PsPAX2 (2.2 μg; #12260; Addgene, Watertown, USA), pMD2.G (3 μg; #12259; Addgene) into cultured 293T cells via calcium phosphate precipitation. After 48 h of incubation, the packaged lentivirus collected from 293T cells was transfected into RAW264.7 cells using Lipofectamine 3000 (#L3000075; Invitrogen) for 48 h.

### Flow cytometry

The cells in the logarithmic growth phase were inoculated into 6-well plates, and the cell suspension was collected after the adherent cells were digested, centrifuged to obtain a cell pellet, mixed with FITC and PI dyes according to the instructions of the Annexin V-FITC/PI cell apoptosis detection kit (#40302ES60; Yeasen, Shanghai, China), and then tested for apoptosis by flow cytometry.

### Bioinformatics

WNT4 is predicted as a potential target of transcription factor ATF3 on the hTFtarget platform ( https://guolab.wchscu.cn/hTFtarget/), which is a comprehensive database for exploring human TF-target regulations. The binding sites between WNT4 promoter and ATF3 were also obtained from this platform.

### ChIP assay

The cells were fixed with 1% formaldehyde for 10 min, and the chromatin was digested to lengths between 150 and 900 base pairs by micrococcal nuclease (#D7201S; Beyotime, Shanghai, China). The cell lysates were immunoprecipitated with an anti-ATF3 antibody or a negative control IgG antibody at 4°C overnight. To purify the bound DNA fragments, the samples were incubated with sodium chloride, heated at 65°C for 4 h and then treated with protease K at 45°C for 60 min. Enrichment of the ATF3 protein with the WNT4 promoter was detected using ChIP-PCR amplification primers for WNT4. The primer and protein information is shown in Supplementary Table S1 and 2.

### Luciferase assay

The NC or AFT3, control or WNT4 promoter luciferase reporter vectors were co-transfected using Lipofectamine 3000 Transfection Reagent (#L3000075; Invitrogen). The cells were lysed 24 h after transfection, and the luciferase activity in the lysate was measured using a GloMax 20/20 (Promega, Madison, USA).

### Alkaline phosphatase (ALP) staining

ATF3 overexpression or interference lentivirus was transfected into RAW264.7 cells. RAW264.7 cells were cultured for 24 h after LPS stimulation, and the supernatant was collected and added to hDPSCs. hDPSCs were inoculated into 48-well culture plates (5 × 10 ^4^ cells/well). After 5 days of induction, the cells were fixed, washed, incubated with ALP staining solution (#ab242286; Abcam, Cambridge, UK) for 0.5 h, rinsed with PBS and observed under a microscope. ImageJ software was used to quantify the staining intensity.

### Alizarin red S (ARS) staining

hDPSCs were inoculated into 48-well culture plates (5 × 10 ^4^ cells/well), cultured for 14 days and then fixed. After being washed, they were stained with 2% ARS staining solution (#A5533; Sigma-Aldrich) at room temperature for 10 min. After being washed, the cells were observed using a digital camera (Carl Zeiss, Wetzlar, Germany), and the staining intensity was quantified via ImageJ.

### Statistical analysis

Statistical analyses were performed using SPSS 21.0 or GraphPad Prism 8. Student’s *t* test and one-way ANOVA were used for analyses between two groups or among multiple groups. The data are expressed as the mean ± standard deviation (SD) from at least three independent experiments. Two-sided tests were performed, and *P* value less than 0.05 was considered to indicate a significant difference.

## Results

### ATF3 expression is low in inflamed pulp tissue

**Figure 1:**
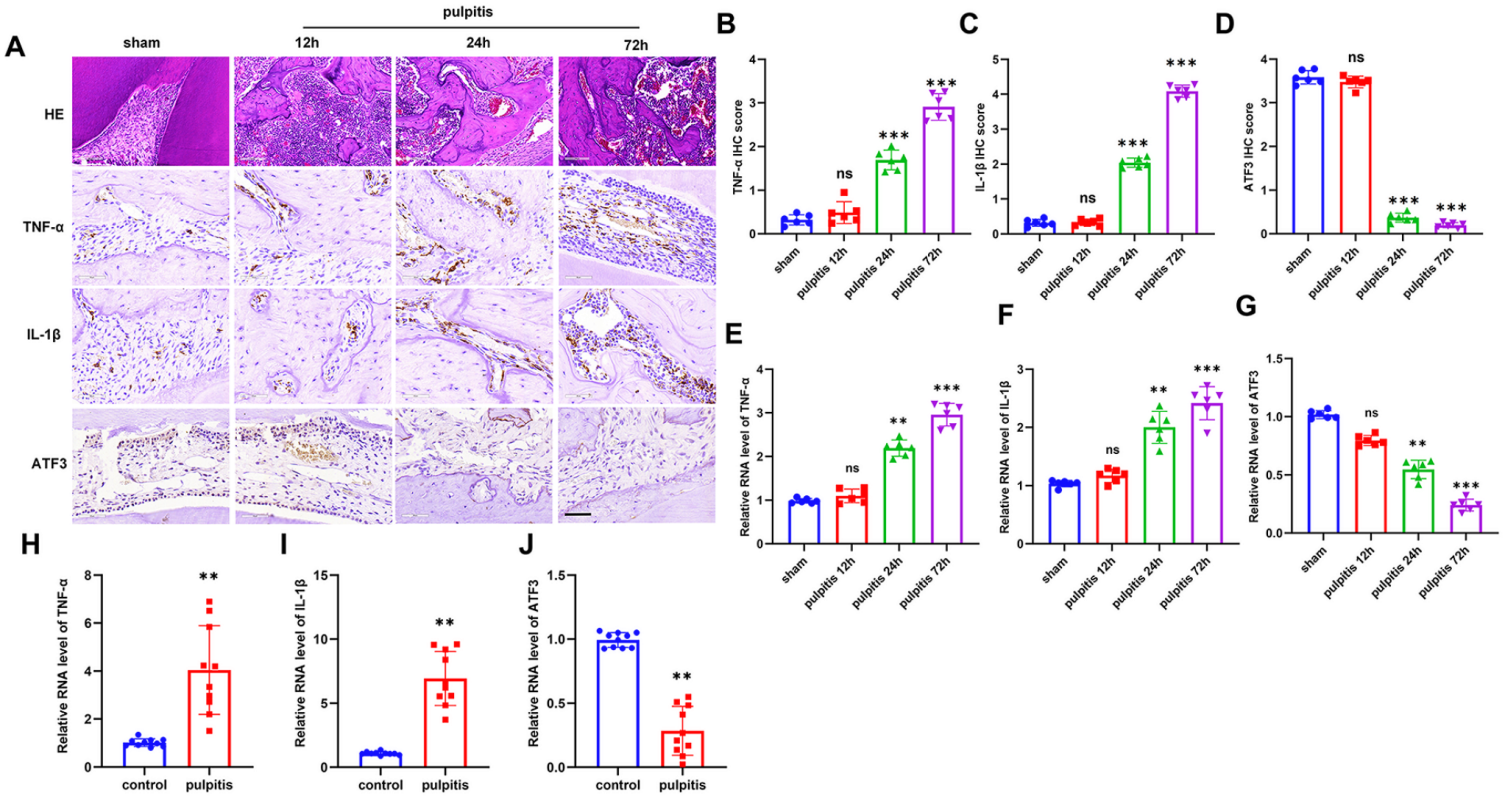
Figure 1 ATF3 is expressed at low levels in inflamed pulp tissue (A) HE staining and IHC staining for TNF-α, IL-1β, and ATF3 in mouse dental pulp tissue from the healthy control group and pulpitis group after 12, 24, and 72 h of pulp exposure (n = 6). Scale bar: 100 μm. (B–D) Statistics of the IHC results for (B) TNF-α, (C) IL-1β, and (D) ATF3 are shown. (E–G) qRT-PCR was performed to detect the mRNA expression levels of (E) TNF-α, (F) IL-1β, and (G) ATF3 in pulp tissues at 12, 24, and 72 h after pulp exposure. (H–J) qRT-PCR was performed to detect the mRNA expression levels of the inflammatory genes (H) TNF-α, (I) IL-1β and (J) ATF3 in inflamed human pulp tissue (n = 10). ns, non-significant. **P < 0.01, ***P < 0.001.

A previous study reported that ATF3 promotes macrophage migration and reverses M1 polarization to the M2 phenotype [12]. To investigate whether ATF3 has the same mechanism of action in pulpitis, we first explored the expression pattern of ATF3 in pulpitis. Experimental pulpitis models with different degrees of inflammation were constructed by exposing mouse pulp for 12, 24, and 72 h. HE staining was used to analyze the histological features of healthy pulp and pulpitis. The results revealed that the normal structure of the inflamed pulp tissue was destroyed, accompanied by the infiltration of many inflammatory cells, vasodilatation and exudation of red blood cells. Pulpitis pathology clearly occurred after 24 h of pulp exposure, and the longer the exposure time was, the more obvious the symptoms were ( Figure 1A). To further clarify the inflammatory state of the dental pulp, classical inflammatory factors were detected by IHC, and the proportions of TNF-α- and IL-1β-positive cells increased in a time-dependent manner after pulp exposure ( Figure 1B,C). Moreover, we found that ATF3-positive cells were abundantly expressed in normal pulp tissues, and the more severe the inflammation in pulpitis was, the lower the number of ATF3-positive cells ( Figure 1D). The above conclusion was further verified by qRT-PCR analysis of inflamed pulp tissues from both mice and humans, which revealed that ATF3 was expressed at low levels in inflamed pulp tissues ( Figure 1E–J).

### ATF3 overexpression alleviates inflammation and macrophage polarization in experimental pulpitis

**Figure 2:**
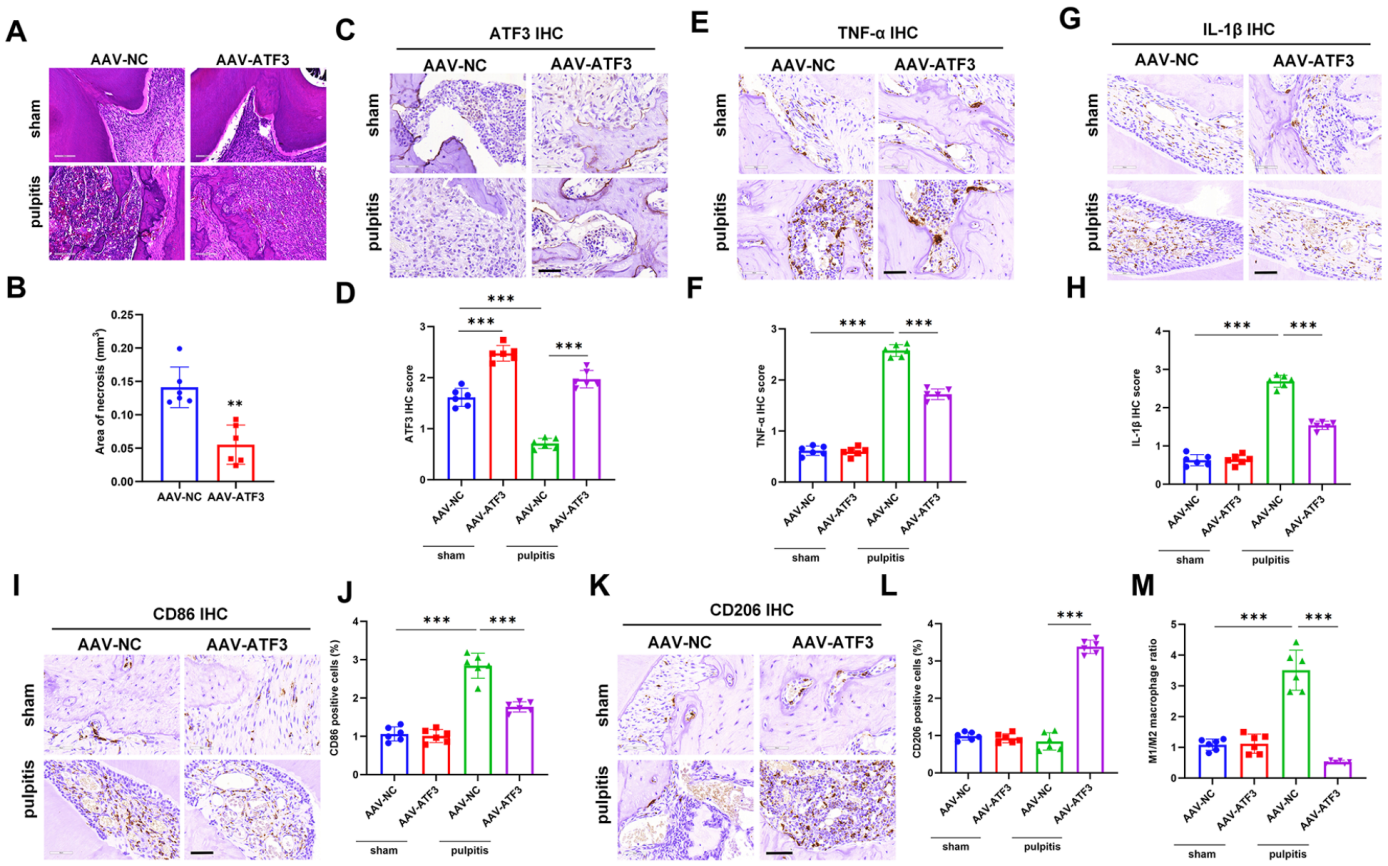
Figure 2 ATF3 overexpression alleviates inflammation and macrophage polarization in experimental pulpitis (A) Schematic diagram of HE staining of mouse dental pulp tissue from the sham and pulpitis groups with or without ATF3 overexpression. Scale bar: 100 μm. (B) Statistics of necrotic areas in dental pulp tissues. (C–H) IHC staining and quantitative analysis of (C,D) ATF3, (E,F) TNF-α, and (G,H) IL-1βexpressions. Scale bar: 100 μm. (I–L) IHC staining and quantitative analysis of the M1 macrophage marker (I,J) CD86 and (K,L) M2 macrophage marker CD206. Scale bar: 100 μm. (M) Ratio of M1/M2 macrophages. **P < 0.01, ***P < 0.001.

An experimental pulpitis mouse model was established, and HE staining revealed that ATF3 overexpression reduced the area of pulp necrosis ( Figure 2A,B). Further IHC staining of TNF-α, IL-1β, and ATF3 in dental pulp tissues revealed that ATF3 overexpression resulted in an increase in the number of ATF3-positive cells in dental pulp tissues and a significant decrease in the number of TNF-α- and IL-1β-positive cells ( Figure 2C–H). To investigate whether ATF3 promotes macrophage migration in pulpitis, we performed IHC analysis of the M1 macrophage marker CD86 and the M2 macrophage marker CD206. ATF3 overexpression decreased the proportion of CD86 ^+^ cells and increased that of CD206 ^+^ cells, and the ratio of M1/M2 macrophages decreased in response to ATF3 overexpression ( Figure 2I–M).

### ATF3 regulates macrophage polarization and inflammation *in vitro*

**Figure 3:**
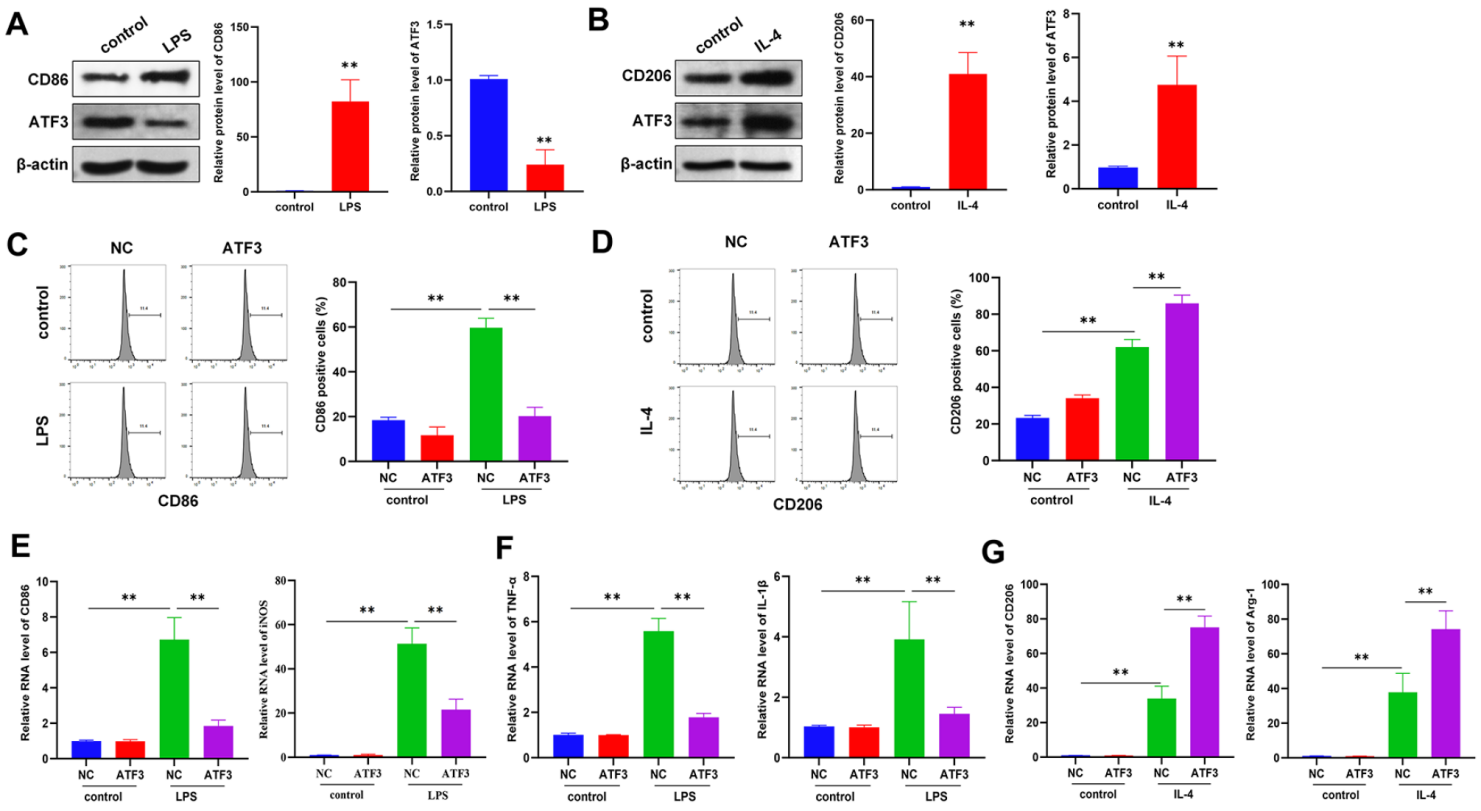
Figure 3 ATF3 regulates macrophage polarization and inflammation *in vitro* (A) Western blot analysis was used to detect the protein expressions of CD86 and ATF3 in macrophages treated with or without 100 ng/mL LPS for 24 h. (B) Western blot analysis of CD206 and ATF3 protein expressions in macrophages treated with or without 10 ng/mL IL-4 for 24 h. (C) The proportion of M1 macrophages was analyzed by flow cytometry after LPS stimulation for 24 h. (D) Flow cytometry analysis was used to evaluate the proportion of M1 macrophages after IL-4 stimulation for 24 h. (E) Expressions of CD86 and iNOS (M1 macrophage markers) in macrophages treated with or without LPS for 24 h. (F) Expressions of the inflammatory factors TNF-α and IL-1β in macrophages treated with or without LPS for 24 h. (G) Expressions of CD206 and Arg-1 (M2 macrophage markers) in macrophages treated with or without IL-4 for 24 h. ** P < 0.01.

An *in vitro* macrophage polarization model was further constructed to investigate the mechanism of action of ATF3. LPS (100 ng/mL) was used to treat RAW264.7 macrophages for 24 h to induce their polarization into M1 macrophages, with untreated RAW264.7 macrophages used as the control. qRT-PCR analysis revealed that ATF3 was expressed at low levels in M1 macrophages ( Figure 3A). Then, we used 10 ng/mL IL-4 to induce the polarization of RAW264.7 cells into M2 macrophages. qRT-PCR analysis revealed that ATF3 was highly expressed in M2 macrophages ( Figure 3B). The overexpression of ATF3 reversed the LPS-induced increase in the proportion of CD86-positive cells and increased the IL-4-induced increase in the number of CD206-positive cells ( Figure 3C,D). Consistently, overexpression of ATF3 downregulated the expressions of the M1 markers CD86 and iNOS and the inflammatory genes TNF-α and IL-1β in LPS-induced M1 macrophages ( Figure 3E,F). In IL-4-induced M2 macrophages, the overexpression of ATF3 further increased the expression of the M2 markers CD206 and Arg-1 ( Figure 3G). Overall, ATF3 promotes polarization from the M1 to the M2 phenotype and reduces the level of inflammation.

### ATF3 regulates WNT4 transcription

**Figure 4:**
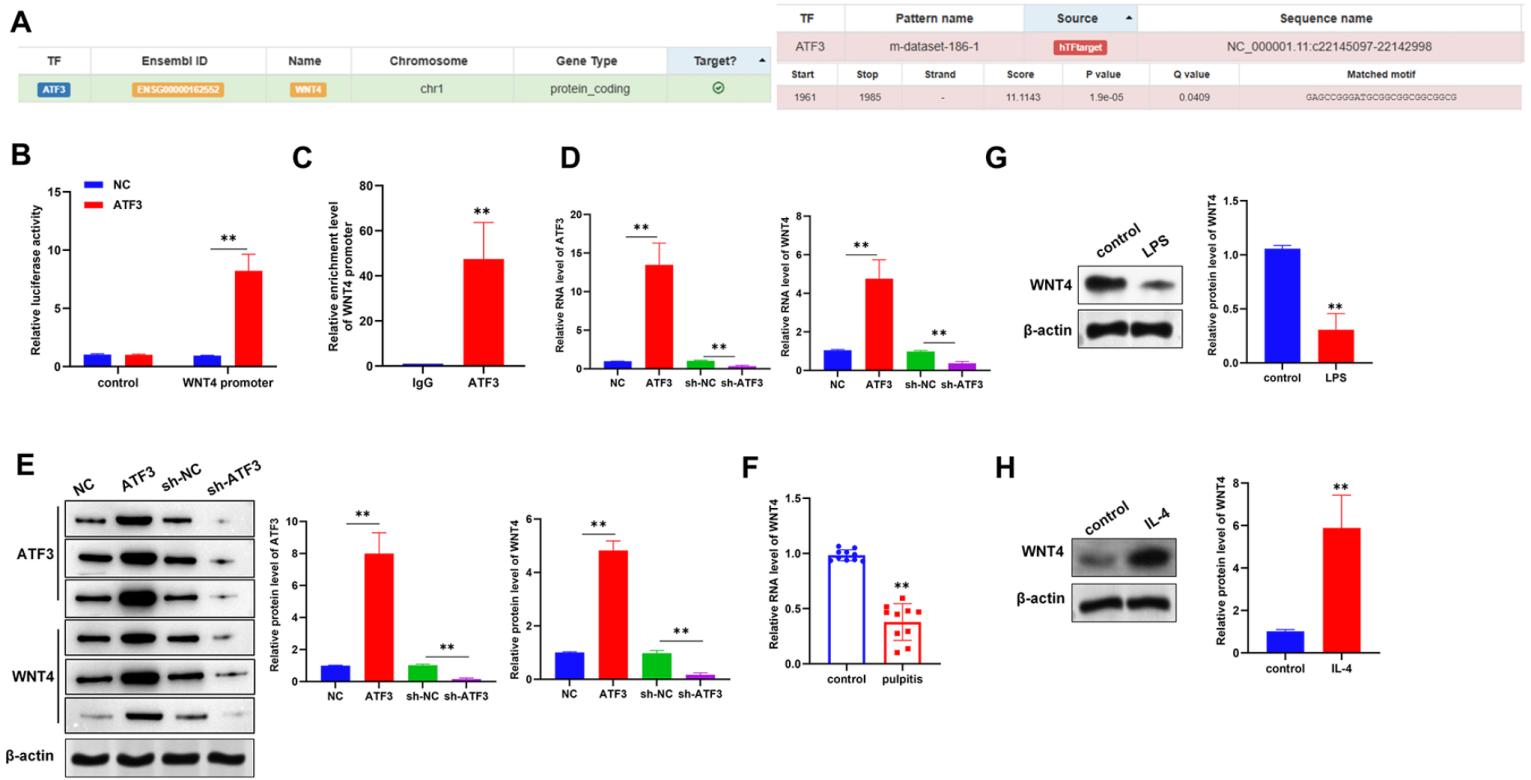
Figure 4 ATF3 regulates WNT4 transcription (A) hTFtargets WNT4 as the target of ATF3. (B) Luciferase reporter assays were conducted to explore the binding between the site of action of ATF3 and the WNT4 promoter. (C) ChIP assays were used to analyze the interaction of ATF3 at the WNT4 promoter. (D,E) qRT-PCR (D) and western blot (E) analysis of WNT4 mRNA and protein levels after knockdown or overexpression of ATF3 in macrophages. (F) qRT-PCR was used to detect the mRNA expression level of WNT4 in inflamed human pulp tissue (n = 10). (G) Western blot analysis was conducted to evaluate WNT4 expression in the untreated control group and the LPS group (100 ng/mL, 24 h). (H) Western blot analysis was used to evaluate WNT4 expression in macrophages in the control and IL-4 groups. Macrophage RAW264.7 cells were treated with 10 ng/mL IL-4 for 24 h to induce M2 polarization in the IL-4 group. **P < 0.01.

A previous study revealed that WNT4 inhibits the pulpal inflammatory response [13]. As predicted by the hTFtarget platform, WNT4 was also one of the predicted targets of the transcription factor ATF3 and was chosen for further analysis. The binding sequence was GAGCCGGGATGCGGCGGCGGCGGCG. ( Figure 4A). The results of the luciferase reporter assays revealed that ATF3 enhanced the fluorescence signal of the WNT4 promoter ( Figure 4B), and the ChIP assay revealed that ATF3 bound to the WNT4 promoter ( Figure 4C). These results revealed that ATF3 can bind to the promoter region of WNT4. We further explored the regulatory role of ATF3 on WNT4, and qRT-PCR and western blot analyses revealed that ATF3 positively regulated the expression of WNT4 ( Figure 4D,E). WNT4 was also expressed at low levels in inflamed human pulp tissues ( Figure 4F). Moreover, we found that WNT4 was expressed at low levels in pro-inflammatory M1 macrophages and at high levels in pro-inflammatory M2 macrophages ( Figure 4G,H).

### ATF3 inhibits macrophage inflammatory responses via WNT4

**Figure 5:**
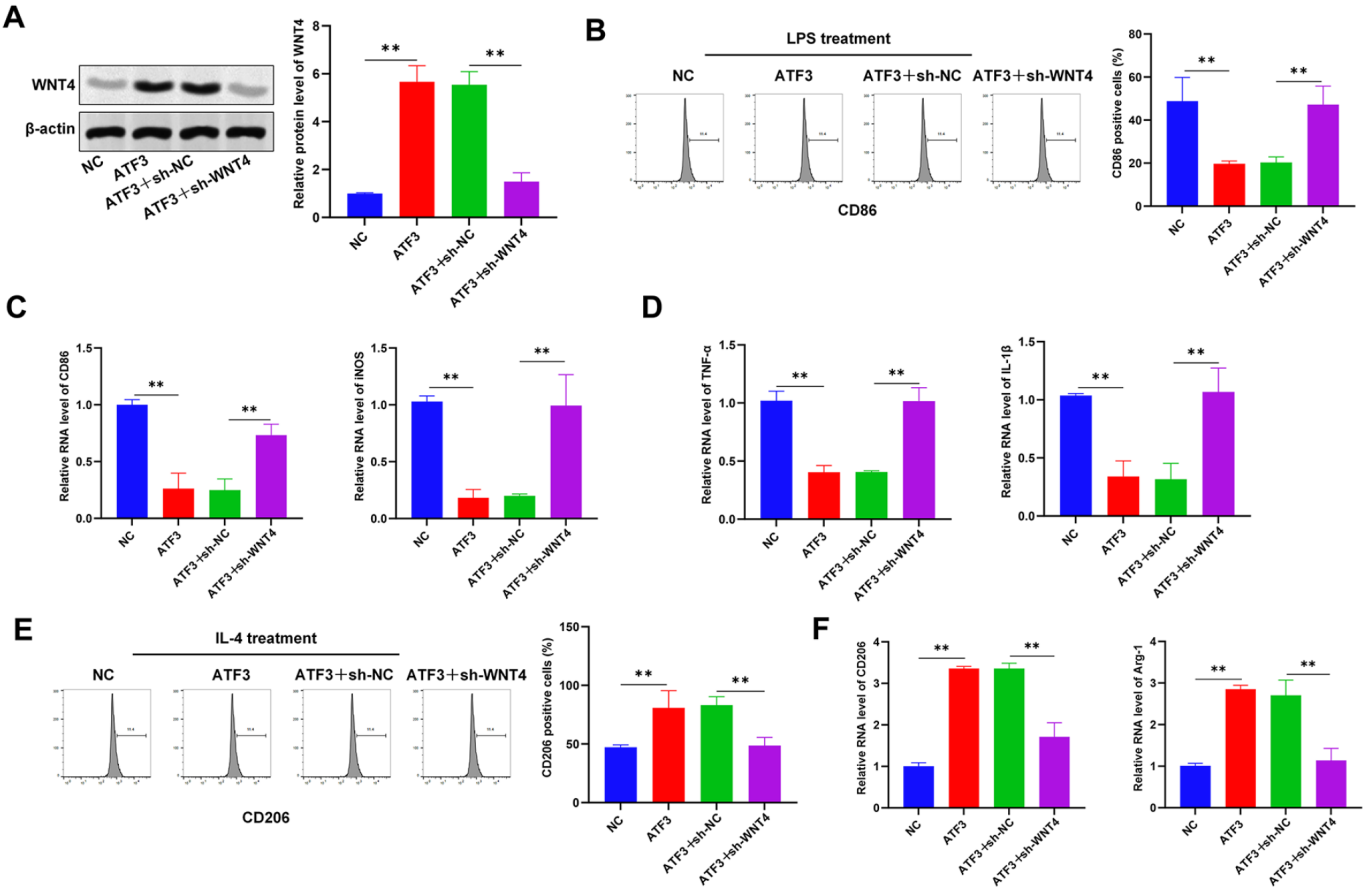
Figure 5 ATF3 inhibits macrophage inflammatory responses via WNT4 Western blot analysis was used to detect the protein levels of WNT4 in macrophages after ATF3 overexpression or WNT4 knockdown. (B) The proportion of M1 macrophages was analyzed by flow cytometry after LPS stimulation for 24 h. (C) Expressions of CD86 and iNOS (M1 macrophage markers) in M1 macrophages after LPS stimulation for 24 h. (D) Expressions of the inflammatory genes TNF-α and IL-1β in M1 macrophages after LPS stimulation for 24 h. (E) Flow cytometry analysis was used to determine the proportion of M1 macrophages after IL-4 stimulation for 24 h. (F) Expressions of CD206 and Arg-1 (M2 macrophage markers) in M2 macrophages after IL-4 stimulation for 24 h. ** P < 0.01.

We further investigated whether ATF3 regulated the inflammatory response in RAW264.7 cells via WNT4. WNT4 in RAW264.7 cells increased in response to ATF3 overexpression but decreased after *WNT4* knockdown, indicating that WNT4 was targeted by ATF3 in RAW264.7 cells ( Figure 5A). In LP-induced M1 pro-inflammatory macrophages, knockdown of *WNT4* reversed the ATF3 overexpression-induced decrease in the expression of the M1 markers CD86 and iNOS and inflammatory factors such as TNF-α and IL-1β ( Figure 5B–D). In IL-4-induced M2 anti-inflammatory macrophages, knockdown of WNT4 reversed the elevated expression of the M2 markers CD206 and Arg-1 induced by ATF3 overexpression ( Figure 5E,F). Overall, ATF3 inhibits the inflammatory response of macrophages by positively regulating WNT4.

### ATF3 promotes the osteogenic differentiation of hDPSCs in an *in vitro* inflammatory microenvironment

**Figure 6:**
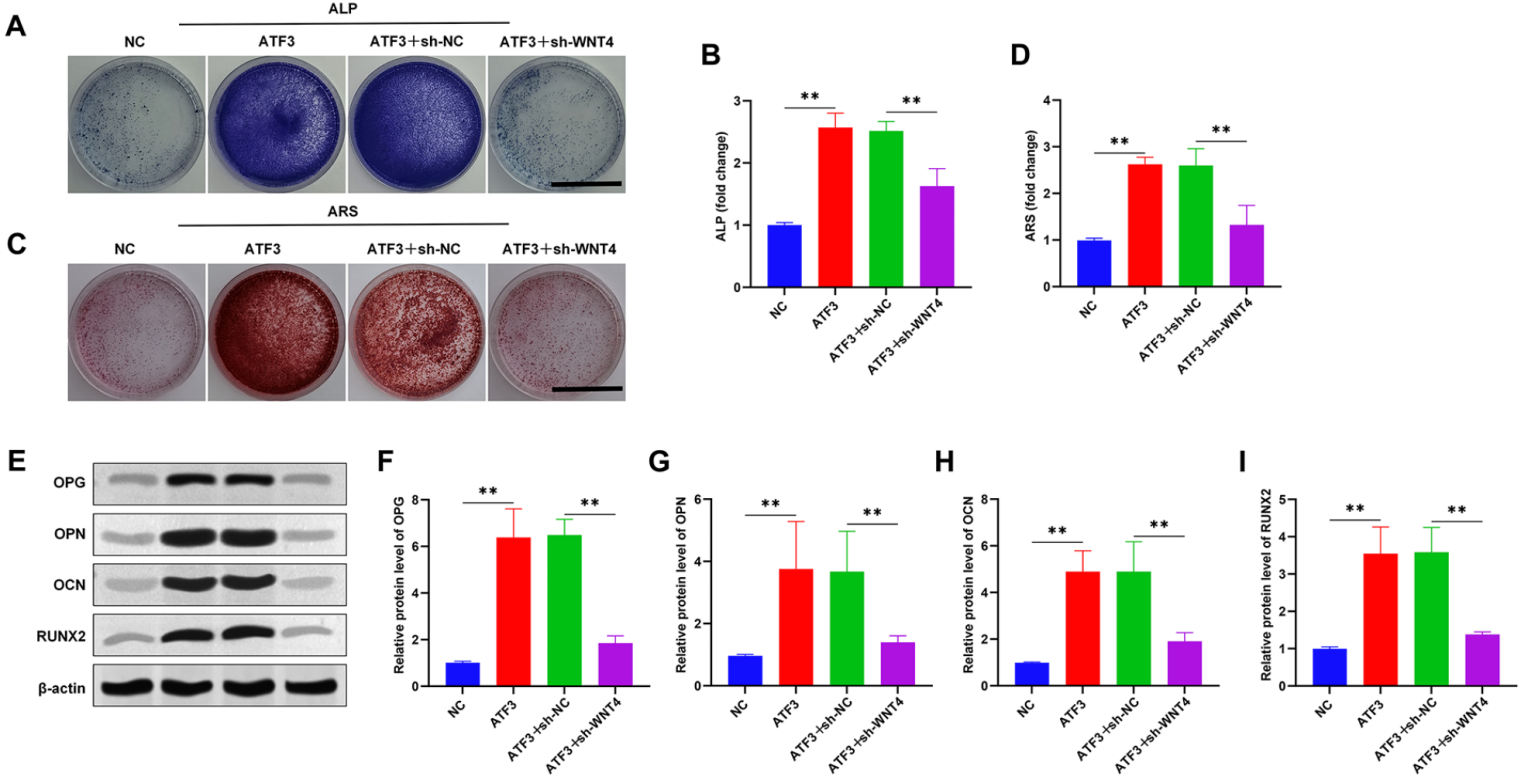
Figure 6 ATF3 promotes the osteogenic differentiation of hDPSCs in an *in vitro* inflammatory microenvironment (A,B) Schematic and quantitative analysis of alkaline phosphatase (ALP) staining. Scale bar: 10 mm. (C,D) Alizarin red (ARS) staining was used to observe and quantify the formation of mineralized nodules. Scale bar: 10 mm. (E–I) Western blot analysis of the protein expression levels of the osteogenesis-related proteins OPG, OPN, OCN and Runx2. **P < 0.01.

A previous study demonstrated that the conversion of the macrophage anti-inflammatory phenotype modulates the osteogenic potential of hDPSCs [14]. ALP and ARS staining were performed to investigate the effects of ATF3 on the osteogenic differentiation of hDPSCs. We found that ATF3 decreased the staining effect of ALP, which was reversed by the knockdown of *WNT4* ( Figure 6A,B). In terms of changes in mineralized nodules, knockdown of WNT4 reversed the ATF3-induced increase in calcified nodules ( Figure 6C,D). Furthermore, the levels of the osteogenesis-related proteins OPG, OPN, OCN, and Runx2 were examined, and knockdown of WNT4 reversed the increase in protein levels caused by ATF3 overexpression ( Figure 6E–I). Overall, ATF3 further promotes the osteogenic differentiation of hDPSCs by regulating WNT4 in the inflammatory microenvironment.

## Discussion

Pulp tissue is the only fibrous loose connective tissue rich in blood vessels, lymphatics and nerves in the tooth structure; it is connected to the whole body through the apical region and possesses a peripheral immune system component [15]. When the pulp becomes infected, microbial stimuli induce a progressive inflammatory response within the pulp, resulting in the development of pulpitis, which is characterized mainly by pain, tissue necrosis and abscesses [ 16, 17] . Reversible pulpitis repairs pulp tissue if inflammation is controlled before pulp necrosis develops [18]. Since the treatment of pulpitis is often accompanied by some degree of adverse effects, it is necessary to explore the pathogenesis and therapeutic targets of pulpitis.

In the present study, we identified ATF3 as a potential therapeutic target for treating endodontitis. ATF3 is a member of the ATF/cAMP response element binding family, which is involved in a variety of cellular processes, including metabolism, the immune response, cell cycle regulation, and inflammatory diseases [19]. ATF3, a common stress sensor, is involved in iron death induction in a variety of diseases, with therapeutic effects on the progression of gastric cancer [20], glioma [21], rectal cancer [22], and other diseases. ATF3 also plays an important role as a marker of neurological injury in neuronal protection and neuroinflammation [23]. Some scholars have analyzed the gene expression profile of dental pulp cells under high glucose conditions and reported that ATF3 is a differentially expressed gene [24]. Therefore, we speculated that ATF3 might be involved in the progression of pulpitis, and surprisingly, we detected low expression of ATF3 in both experimental pulpitis models and human pulpitis samples. Furthermore, ATF3 was found to drive macrophage polarization toward an anti-inflammatory phenotype through the Wnt/β-catenin signaling pathway [12]. Local accumulation of inflammatory mediators, which are characteristic of endodontitis, further links ATF3, endodontitis and inflammation.

As essential members of the intrinsic immune system, macrophages play important regulatory roles in the phagocytosis of pathogenic periodontal microorganisms and the generation of inflammation [25]. Numerous studies have shown that macrophage infiltration is increased in diseased periodontal tissues and that the proportion of infiltrating inflammatory cells is as high as 30%, suggesting that endodontitis is closely related to macrophages [ 11, 26] . Under the action of Th2 cells and local stimulating factors, macrophages differentiate into M2-type macrophages and play an important role in tissue repair in pulpitis by promoting local tissue repair and regeneration and maintaining homeostasis [27]. In this study, we found that the overexpression of ATF3 decreased the number of M1 macrophages and pro-inflammatory factors and increased the number of M2 macrophages, suggesting that the overexpression of ATF3 may alleviate the inflammatory response by promoting M2 polarization. It is well known that TNF-α and IL-6 are typical pro-inflammatory factors that are involved in the regulation of local immune responses. They are produced in the acute phase of inflammation and promote the conversion of neutrophils to monocytes/macrophages, which can control the transition of inflammation from acute to chronic. In pulpitis, pro-inflammatory factors are involved in the development of inflammation and are generally expressed at high levels [28]. To explore the mechanism of action of ATF3 in vitro, we induced RAW264.7 cells with LPS and IL-4 to generate M1-polarized and M2-polarized states, respectively. LPS is one of the most potent activators of the human innate immune system and is commonly used to stimulate the inflammatory response in endodontic inflammation [29]. The results revealed that overexpression of ATF3 suppressed the number of M1 macrophages and increased the number of M2 macrophages, which was consistent with our *in vivo* experiments and conclusions.

Further exploration of the pathway of action of ATF3 revealed that WNT4 was its downstream target. It has been demonstrated that WNT4 inhibits the pulpal inflammatory response [13]. Therefore, we hypothesized that ATF3 positively regulates WNT4 and inhibits the progression of pulpitis. In this study, we found that WNT4 was expressed at low levels in the M1-polarized state and highly expressed in the M2-polarized state. In addition, knocking down WNT4 reversed the promotion of M2 polarization caused by ATF3 overexpression and the inhibition of pro-inflammatory factors. It is well known that LPS induces the production of many cytokines, including TNF-α, IL-6 and IL-1β [30]. In this study, we examined the expression of the cytokines TNF-α, IL-6 and IL-1β to determine the degree of inflammation and pathological state of endodontitis. In summary, we conclude that ATF3 promotes the polarization of M2 macrophages and thus alleviates the inflammatory response by positively regulating WNT4. In addition, we found that ATF3 promoted the osteogenic differentiation of hDPSCs, suggesting that the overexpression of ATF3 promotes pulp mineralization and facilitates pulp regeneration and repair.

**Figure 7:**
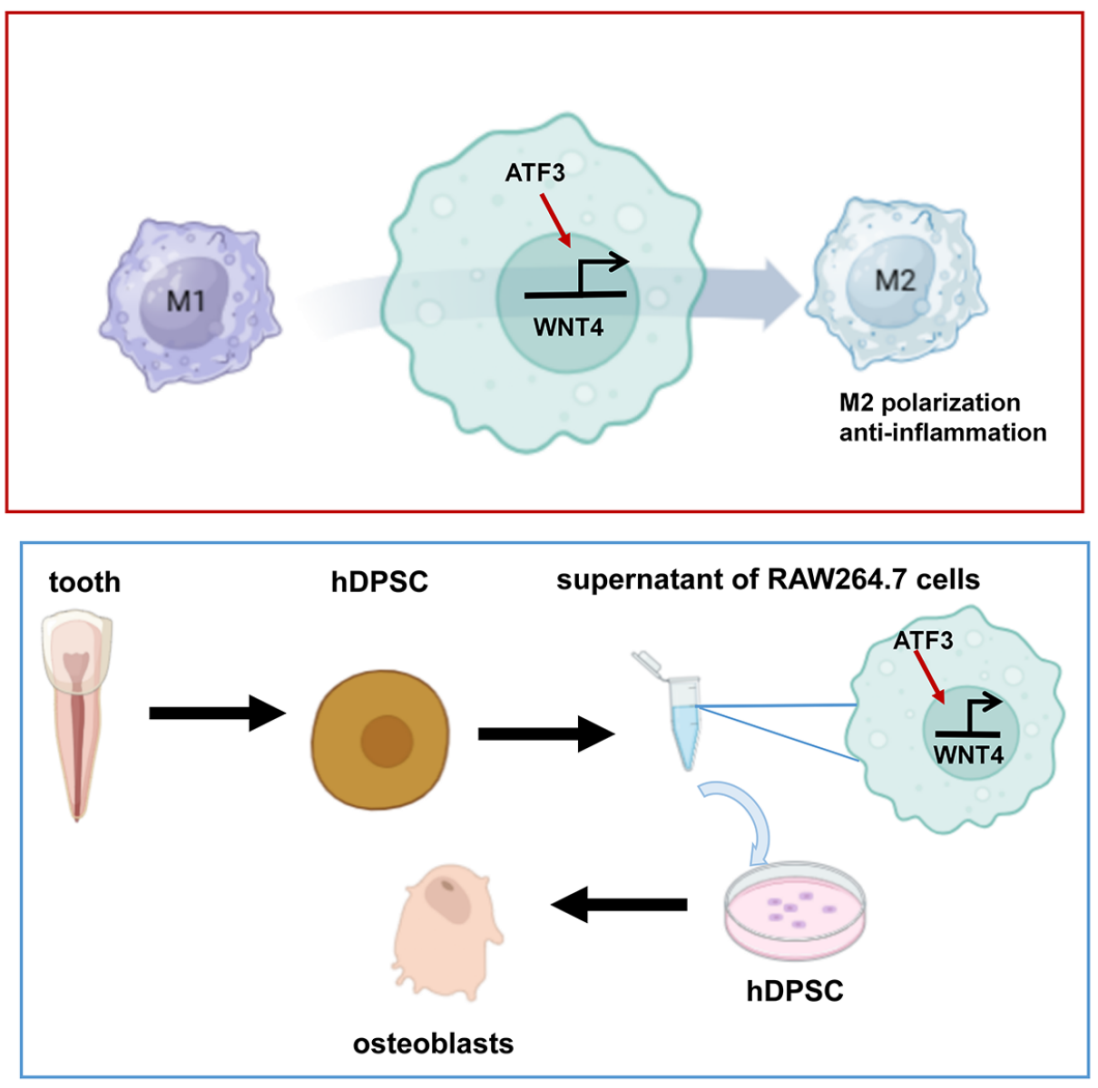
Figure 7 A schematic diagram of the molecular mechanism of ATF3 in pulp inflammation ATF3 facilitates M2 macrophage polarization by promoting WNT4 transcription, which in turn inhibits inflammatory responses and promotes the osteogenic differentiation of human dental pulp stem cells.

However, there are still several limitations in this study. First, the effects of ATF3 on dental pulp mineralization and repair were not explored in animal experiments, although this does not affect our main conclusion in this study. Despite this limitation, our study revealed that ATF3 promotes M2 macrophage polarization via WNT4, which in turn inhibits inflammatory responses and promotes the osteogenic differentiation of human dental pulp stem cells ( Figure 7).

## Supporting information

24747Supplementary_Table_1

24747Supplementary_Table_3

24747Supplementary_Table_2

## Supplementary Data

Supplementary data is available at *Acta Biochimica et Biophysica Sinica* online.

## References

[1] Park SH, Ye L, Love RM, Farges JC, Yumoto H (2015). Inflammation of the dental pulp. Mediators Inflamm.

[2] Dye B, Thornton-Evans G, Li X, and Iafolla T. Dental caries and tooth loss in adults in the United States, 2011–2012.
*
NCHS Data Brief
* 2015, 197: 197. https://pubmed.ncbi.nlm.nih.gov/25973996/.

[3] Zanini M, Meyer E, Simon S (2017). Pulp inflammation diagnosis from clinical to inflammatory mediators: a systematic review. J Endods.

[4] Galler KM, Weber M, Korkmaz Y, Widbiller M, Feuerer M (2021). Inflammatory response mechanisms of the dentine-pulp complex and the periapical tissues. Int J Mol Sci.

[5] Farges JC, Alliot-Licht B, Renard E, Ducret M, Gaudin A, Smith AJ, Cooper PR (2015). Dental pulp defence and repair mechanisms in dental caries. Mediators Inflamm.

[6] Koca-Ünsal RB, Şehirli AÖ, Sayıner S, Aksoy U (2022). Relationship of NLRP3 inflammasome with periodontal, endodontic and related systemic diseases. Mol Biol Rep.

[7] Sarfi S, Azaryan E, Naseri M (2024). Immune system of dental pulp in inflamed and normal tissue. DNA Cell Biol.

[8] Luo M, Zhao F, Cheng H, Su M, Wang Y (2024). Macrophage polarization: an important role in inflammatory diseases. Front Immunol.

[9] Yunna C, Mengru H, Lei W, Weidong C (2020). Macrophage M1/M2 polarization. Eur J Pharmacol.

[10] Locati M, Curtale G, Mantovani A (2020). Diversity, mechanisms, and significance of macrophage plasticity. Annu Rev Pathol Mech Dis.

[11] Le Fournis C, Jeanneau C, Giraud T, El Karim I, Lundy FT, About I (2021). Fibroblasts control macrophage differentiation during pulp inflammation. J Endods.

[12] Sha H, Zhang D, Zhang Y, Wen Y, Wang Y (2017). ATF3 promotes migration and M1/M2 polarization of macrophages by activating tenascin-C via Wnt/β-catenin pathway. Mol Med Rep.

[13] Ni C, Wu G, Miao T, Xu J (2022). Wnt4 prevents apoptosis and inflammation of dental pulp cells induced by LPS by inhibiting the IKK/NF‑κB pathway. Exp Ther Med.

[14] Liu B, Li J, Chen B, Shuai Y, He X, Liu K, He M (2023). Dental pulp stem cells induce anti-inflammatory phenotypic transformation of macrophages to enhance osteogenic potential via IL-6/GP130/STAT3 signaling. Ann Transl Med.

[15] Rombouts C, Giraud T, Jeanneau C, About I (2017). Pulp vascularization during tooth development, regeneration, and therapy. J Dent Res.

[16] Siqueira JF, Rôças IN (2022). Present status and future directions: microbiology of endodontic infections. Int Endod J.

[17] Wang Y, Chen W, Hao L, McVicar A, Wu J, Gao N, Liu Y (2019). C1 silencing attenuates inflammation and alveolar bone resorption in endodontic disease. J Endods.

[18] Bjørndal L, Simon S, Tomson PL, Duncan HF (2019). Management of deep caries and the exposed pulp. Int Endod J.

[19] Ku HC, Cheng CF (2020). Master regulator activating transcription factor 3 (ATF3) in metabolic homeostasis and cancer. Front Endocrinol.

[20] Fu D, Wang C, Yu L, Yu R (2021). Induction of ferroptosis by ATF3 elevation alleviates cisplatin resistance in gastric cancer by restraining Nrf2/Keap1/xCT signaling. Cell Mol Biol Lett.

[21] Lu S, Wang X, He C, Wang L, Liang S, Wang C, Li C (2021). ATF3 contributes to brucine-triggered glioma cell ferroptosis via promotion of hydrogen peroxide and iron. Acta Pharmacol Sin.

[22] Liu J, Lu X, Zeng S, Fu R, Wang X, Luo L, Huang T (2024). ATF3-CBS signaling axis coordinates ferroptosis and tumorigenesis in colorectal cancer. Redox Biol.

[23] Zhang Y, Xu T, Xie J, Wu H, Hu W, Yuan X (2024). MSC-derived mitochondria promote axonal regeneration via Atf3 gene up-regulation by ROS induced DNA double strand breaks at transcription initiation region. Cell Commun Signal.

[24] Horsophonphong S, Sritanaudomchai H, Nakornchai S, Kitkumthorn N, Surarit R (2021). Odontogenic gene expression profile of human dental pulp-derived cells under high glucose influence: a microarray analysis. J Appl Oral Sci.

[25] Song X, Xue Y, Fan S, Hao J, Deng R (2022). Lipopolysaccharide-activated macrophages regulate the osteogenic differentiation of bone marrow mesenchymal stem cells through exosomes. Peer J.

[26] Wang TT, Jiang WR, Xu L, Zhou MY, Huang YS (2024). Effect of blockage of Trem1 on the M1 polarization of macrophages in the regulation dental pulp inflammation. Eur J Oral Sci.

[27] Li J, Ren H, Zhang Z, Zhang J, Wei F (2024). MacrophageM2 polarization promotes pulpal inflammation resolution during orthodontic tooth movement. J Cell Mol Medi.

[28] Chen J, Xu H, Xia K, Cheng S, Zhang Q (2021). Resolvin E1 accelerates pulp repair by regulating inflammation and stimulating dentin regeneration in dental pulp stem cells. Stem Cell Res Ther.

[29] Kim JH, Irfan M, Hossain MA, Shin S, George A, Chung S (2023). LPS-induced inflammation potentiates dental pulp stem cell odontogenic differentiation through C5aR and p38. Connective Tissue Res.

[30] Reichl FX, Högg C, Liu F, Schwarz M, Teupser D, Hickel R, Bloch W (2020). Actovegin® reduces PMA-induced inflammation on human cells. Eur J Appl Physiol.

